# Dental Age Assessment using Demirjian's Eight Teeth Method and Willems Method in a Tertiary Hospital

**DOI:** 10.31729/jnma.3876

**Published:** 2018-12-31

**Authors:** Nitin Kumar Agrawal, Lucina Hackman, Samarika Dahal

**Affiliations:** 1 Department of Dentistry, Institute of Medicine, Maharajgunj, Kathmandu, Nepal; 2 Centre for Anatomy and Human Identification, University of Dundee, Dundee, United Kingdom

**Keywords:** *dental*, *forensic anthropology*, *forensic dentistry*, *Nepal*, *odontology*

## Abstract

**Introduction:**

Age estimation is an important aspect in forensic anthropology, as it can aid in the identification of the deceased, and can be used in cases of immigration, child abuse and criminal prosecution in living individuals. Dental age estimation is considered reliable and accurate, since tooth development is least affected by environmental factors compared to somatic growth.

**Methods:**

In total, 150 pre-orthodontic treatment radiographs from healthy individuals were assessed. These individuals were aged between 8 to 19 years. Dental age for these individuals was calculated by two methods: Demirjian's eight teeth method and Willems method. For Willems method, seven teeth on the left side of mandible (except the third molar) were staged according to Demirjian's staging, and for Demirjian's eight teeth method, all eight teeth were staged.

**Results:**

The mean chronological ages were 13.6961+1.94384 years in males and 13.9204+2.63541 years in females. The mean estimated ages by Demirjian's eight teeth method were 12.1856+1.73478 years and 11.7906+2.32344 years in males and females respectively. Similarly, the mean estimated ages by Willems method were 12.8958+1.46838 years in males and 12.6926+2.27807 years in females.

**Conclusions:**

Willems method and Demirjian's eight teeth method underestimated the chronological age in the given population. Both methods showed excellent correlation with chronological age indicating their applicability in dental age estimation, with development of population specific scores.

## INTRODUCTION

Identification of a deceased individual is essential in terms of legal, ethical, religious and social aspects.^1^ This is undertaken by establishing a biological profile, age estimation being one of the important aspects in this process.^2,3^ The various methods used for age estimation include: development of dentition, closure of cranial sutures, morphological changes in ribs and os-coxae, histomorphometric methods and amino-acid racemization of dentine.^4^ Tooth development is considered an important indicator of age.^5,6^

In order to provide age estimations, which can be applied to a given population, population specific standards are necessary. This has been attributed to developmental variation between different populations; however, exact biological/ socio-geographical/ socio-economic mechanism for these differences is not known.^7^ There is paucity of literature regarding the accuracy of age estimation methods in a Nepalese population.

The main objective of this study was to test the accuracy of Demirjian's eight teeth method^8^ and Willems method^9^ in selected population of a tertiary hospital.

## METHODS

This descriptive cross-sectional study was carried out at the University of Dundee, United Kingdom from April 2016 to August 2016. The study was conducted in accordance with Helsinki Declaration. Convenient sampling was done in this study. The assessment was carried out using digital panoramic radiographs or orthopantomograms of orthodontic patients from Nepal. An orthopantomogram is defined as a radiograph that is taken extra-orally and displays a panoramic view of the entire dentition, alveolar bone, and other adjacent structures on a single film. Inclusion and exclusion criteria were set prior to assessment of the radiographs. Inclusion criteria included orthopantomograms of orthodontic patients and good quality radiographs. Exclusion criteria included the any presence of gross pathology, radiographs of unknown age or sex or the absence of multiple teeth.

In total, 150 radiographs were procured. All radiographs were from healthy individuals and were taken prior to commencement of their orthodontic treatment. No radiographs were taken for the express purposes of this study ensuring that no individual were exposed to ionising radiation unnecessarily.

The investigator performing the dental age assessment was blinded regarding the chronological age of the patient. The chronological age of the patient was calculated by subtracting the date of birth as provided in the dental record, from the date of radiograph taken which was present in the digital radiograph. Radiographs were anonymized at source and the only information collected were sex, date of birth and date of image.

The dental age was calculated using the two methods: Demirjian's eight teeth method^8^ and Willems method.^9^ Dental age obtained by these methods was then compared to the chronological age of the individuals for evaluating their accuracy. Statistical significance level was set to 0.05. For comparison, the data was then entered in IBM SPSS 22.0 and descriptive analysis was done.

## RESULTS

In total 150 orthopantomograms belonging to 60 (40%) males and 90 (60%) females (Table 1). The mean chronological ages for each group were estimated to be 13.9204±2.63541 years for females and 13.6961 ±1.94384 years. After age estimation using Demirjian's eight teeth method, the mean estimated ages were 11.7906±2.32344 years for females, and 12.1856 ± 1.73478 years for males.

**Table 1:** Sample distributions according to age groups.

Age Group	Males	Females
8–12 years	12 (20%)	20 (22.22%)
12–16 years	39 (65%)	43 (47.78%)
16–19 years	9 (15%)	27 (30%)
Total	60	90

Similarly, the mean estimated ages by Willems method were 12.6926±2.27807 for females, and 12.8958±1.46838 for males (Table 2).

**Table 2:** Paired T test for females and males.

		Mean	N	Std. Deviation	Paired Differences		t	df	P
					Mean Difference	Std. Deviation			
Pair 1	Estimated age Demirjian 8 teeth Female	11.7906	90	2.32344	−2.12984	1.34891	−14.979	89	<0.001
	Chronological age female	13.9204	90	2.63541					
Pair 2	Chronological age female	13.9204	90	2.63541	1.22789	1.45061	8.03	89	<0.001
	Estimated Willems female age	12.6926	90	2.27807					
Pair 3	Estimated age Demirjian 8 teeth Female	11.7906	90	2.32344	−0.90195	0.52673	−16.245	89	<0.001
	Estimated Willems female age	12.6926	90	2.27807					
Pair 4	Estimated age Demirjian 8 teeth Male	12.1856	60	1.73478	−1.51046	0.95635	−12.234	59	<0.001
	Chronological age male	13.6961	60	1.94384					
Pair 5	Chronological age male	13.696	60	1.94384	0.80023	1.13413	5.466	59	<0.001
	Estimated Willems Male age	12.8958	60	1.46838					
Pair 6	Estimated age Demirjian 8 teeth Male	12.1856	60	1.73478	−0.71022	0.58064	−9.475	59	<0.001
	Estimated Willems Male age	12.8958	60	1.46838					

On comparison of the mean values of estimated Demirjian's eight teeth method and chronological age, the mean values of chronological age of both sexes were higher than that calculated by Demirjian's eight teeth method. The difference between the means was statistically significant with a P value of <0.001. The findings were similar for Willems method as well. The mean chronological age was higher than the mean estimated age for both males and females, and the difference was statistically significant with a P value of <0.001 (Table 2).

On comparison of error margin for two methods, the difference between chronological age and estimated dental age was higher for Demirjian's eight teeth method than Willems method. The mean differences between both methods for males and females were 0.71022±0.58064 years and 0.94108±0.63519 years respectively (Table 3).

**Table 3:** Comparison of error margin of two methods.

		Mean	N	Std. Deviation	Paired Differences		t	df	P
					Mean Difference	Std. Deviation			
Pair 1	Chronological age - estimated Demirjian age female	2.169	90	1.36218	0.94108	0.63519	14.055	89	<0.001
	Chronological age - estimated Willems age female	1.2279	90	1.45061					
Pair 2	Chronological age - estimated Demirjian age male	1.5105	60	0.95635	0.71022	0.58064	9.475	59	<0.001
	Chronological age - estimated Willems age male	0.8002	60	1.13413					

This showed that Willems method was better than Demirjian's eight teeth method due to lower mean difference in age estimation.

On applying Pearson's correlation, it was observed that there was an excellent correlation between Demirjian's eight teeth method and chronological age in both sexes (0.859 for females; 0.871 for males). There was an excellent correlation between Willems method and chronological age in both males (0.814) and females (0.835) as well. Similarly, there was also an excellent correlation between the two age estimation methods in both sexes (0.974 for females; 0.948 for males). All these correlations were significant with a P value of <0.001.

## DISCUSSION

Tooth development is considered as an important indicator of age.^5,6^ This is due to the fact that tooth development can be classified into several developmental stages as defined by several authors^10,11^ and a tooth passes through the same stages in every individual with each stage indicating the stage of maturity that has been reached by the individual. ^11^ Tooth development can be assessed based on eruption or the process of tooth mineralization.^12,13^ Moreover, teeth being the hardest part of human body can often survive longest in adverse conditions, for example, they may be the only remnant in burnt bodies, thus serving as a reliable element in the identification of a deceased.^14^ Tooth development was used in the identification of two children in one of the air crashes in Nepal.^15^

In this study, the sex of the individuals have been taken into account, as it is considered to affect dental development.^16,17^ Hilgers et al.^18^ hypothesized that the sex specific differences in dental maturation can be accounted for due to hormonal factors. Therefore, males and females were assessed as two separate groups.

In this study, Demirjian's eight teeth method underestimated the ages in both sexes, when compared to the mean chronological age of both males and females. This has been seen in other studies, for example, Demirjian's eight teeth method underestimated the dental age in Indian population^18^ and in South Indian children.^19^ Acharya^18^ reasoned the underestimation of age, due to addition of third molar, which would have contributed to overall slowing down of tooth advancement in Indian population. The same reason can be attributed to the sample in our study, as Nepalese population show similar anthropometric similarity to an Indian population. ^21^

In the present study, Willems method also underestimated age in both sexes and the difference was statistically significant. The underestimation of age by Willems method in our study is in congruence with studies done by other authors.^20,22^

The Willems method due to lower mean difference, showed better performance between the two methods in both the sexes. This could be because of adjustment of scores given by Demirjian et al. ^11^ by Willems et al.^9^ almost after two decades. This would have addressed dental maturation due to secular trend.^13^ Thus, Willems method reflected age more accurately when compared to Demirjian's method.^23^

There were a number of limitations of the study including the facts that ethnicity and other environmental factors like habits, nutrition, and disease could not be considered. The sample size was relatively small for the results to be extrapolated to the whole population, but the accuracy of these methods have not been tested on this population before and this study will go some way to allowing practitioners to choose the best method to use when undertaking age estimation for the dentition on someone from this population.

## CONCLUSIONS

Willems method and Demirjian's eight teeth method underestimated the chronological age but correlated well with the chronological age of the given population. In order to apply Demirjian's eight teeth method and Willems method in Nepalese population, for dental age estimation, we recommend developing baseline data for these methods, which can serve as representative of Nepalese population.
